# Preparation of Fly Ash-Ladle Furnace Slag Blended Geopolymer Foam via Pre-Foaming Method with Polyoxyethylene Alkyether Sulphate Incorporation

**DOI:** 10.3390/ma15124085

**Published:** 2022-06-08

**Authors:** Ng Hui-Teng, Heah Cheng-Yong, Liew Yun-Ming, Mohd Mustafa Al Bakri Abdullah, Catleya Rojviriya, Hasniyati Md Razi, Sebastian Garus, Marcin Nabiałek, Wojciech Sochacki, Ilham Mukriz Zainal Abidin, Ng Yong-Sing, Agata Śliwa, Andrei Victor Sandu

**Affiliations:** 1 Geopolymer and Green Technology, Centre of Excellence (CEGeoGTech), Universiti Malaysia Perlis (UniMAP), Kangar 01000, Perlis, Malaysia; nghuiteng93@gmail.com (N.H.-T.); ymliew@unimap.edu.my (L.Y.-M.); mustafa_albakri@unimap.edu.my (M.M.A.B.A.); nicholas.zai.1130@gmail.com (N.Y.-S.); 2 Faculty of Chemical Engineering Technology, Universiti Malaysia Perlis (UniMAP), Kangar 01000, Perlis, Malaysia; 3 Faculty of Mechanical Engineering Technology, Universiti Malaysia Perlis (UniMAP), Kangar 01000, Perlis, Malaysia; 4 Synchrotron Light Research Institute, Muang, Nakhon Ratchasima 30000, Thailand; catleya@slri.or.th; 5 Malaysian Nuclear Agency, Bangi, Kajang 43000, Selangor, Malaysia; hasniyati@nm.gov.my (H.M.R.); mukriz@nm.gov.my (I.M.Z.A.); 6 Faculty of Mechanical Engineering and Computer Science, Częstochowa University of Technology, 42-201 Częstochowa, Poland; sebastian.garus@pcz.pl (S.G.); wojciech.sochacki@pcz.pl (W.S.); 7 Department of Physics, Częstochowa University of Technology, 42-201 Częstochowa, Poland; marcin.nabialek@pcz.pl; 8 Division of Materials Processing Technology and Computer Techniques in Materials Science, Silesian University of Technology, 44-100 Gliwice, Poland; agata.sliwa@polsl.pl; 9 Faculty of Materials Science and Engineering, “Gheorghe Asachi” Technical University of Iasi, 41 “D. Mangeron” Street, 700050 Iasi, Romania; 10 Romanian Inventors Forum, Str. Sf. P. Movila 3, 700089 Iasi, Romania

**Keywords:** polyoxyethylene alkyether sulphate foam, blended geopolymer foam, high temperature, pre-foaming method

## Abstract

This paper uses polyoxyethylene alkyether sulphate (PAS) to form foam via pre-foaming method, which is then incorporated into geopolymer based on fly ash and ladle furnace slag. In the literature, only PAS-geopolymer foams made with single precursor were studied. Therefore, the performance of fly ash-slag blended geopolymer with and without PAS foam was investigated at 29–1000 °C. Unfoamed geopolymer (G-0) was prepared by a combination of sodium alkali, fly ash and slag. The PAS foam-to-paste ratio was set at 1.0 and 2.0 to prepare geopolymer foam (G-1 and G-2). Foamed geopolymer showed decreased compressive strength (25.1–32.0 MPa for G-1 and 21.5–36.2 MPa for G-2) compared to G-0 (36.9–43.1 MPa) at 29–1000 °C. Nevertheless, when compared to unheated samples, heated G-0 lost compressive strength by 8.7% up to 1000 °C, while the foamed geopolymer gained compressive strength by 68.5% up to 1000 °C. The thermal stability of foamed geopolymer was greatly improved due to the increased porosity, lower thermal conductivity, and incompact microstructure, which helped to reduce pressure during moisture evaporation and resulted in lessened deterioration.

## 1. Introduction

Today, the excellent lightweight and mechanical properties, good thermal and chemical stability, and low environmental impact have led to the successful use of porous geopolymer or geopolymer foam as a catalyst, thermal insulator, adsorbent and filter [1]. Geopolymer foam can be made by mixing aluminosilicate material (natural resource or industrial waste), foaming agent and alkali solution. There are numerous methods that can be used to prepare geopolymer foam, such as replica, sacrificial filler, pre-emulsification, solid impregnation, direct foaming and pre-foaming [1,2,3]. The pre-foaming method involves a two-step process, which is more complex compared to the commonly used direct foaming method, as it only involves a one-step process. In the direct foaming method, the foaming agent is directly added to the geopolymer mixture. In the pre-foaming method, the foaming agent is mixed with water to generate foam through a foam generator, and the generated foam is then added to the geopolymer mixture.

Several studies have been focused on the production of geopolymer foam by adopting the pre-foaming method, with the incorporation of polyoxyethylene alkyether sulphate (PAS) foaming agent. PAS is a brown liquid extracted from coconut oil, and it can be easily and quickly dissolved in water to generate foam [4]. Based on previous studies, Ibrahim et al. [5] produced fly ash geopolymer foam with PAS foam-to-paste ratios ranging from 0.5 to 2.0, revealing that the compressive strength of the foamed geopolymer (17.8–4.1 MPa) decreased with increasing foam-to-paste ratio. Mastura et al. [6] and Ibrahim et al. [4] produced fly ash geopolymer foam with a PAS foam-to-paste ratio of 1.0, reporting that the compressive strength of the foamed geopolymer was in the range of 9.0 MPa to 19.3 MPa. On the other hand, Tiong et al. [7] also produced concrete foam using PAS foam.

The past literature examined the influence of PAS foam addition on geopolymer at room temperature, as well as the influence of PAS foam addition on geopolymer at high temperatures. Mastura et al. [2] studied the effect of high temperature exposure on the strength performance of fly ash geopolymer foam with a PAS foam-to-paste ratio of 1.0. The results showed that the compressive strength of the foamed geopolymer reduced with the increase of temperature from 200 °C (33.3 MPa) to 800 °C (18.6 MPa).

Moreover, in previous studies [8,9], geopolymer foams were mainly based on a single precursor. Very little research has been investigated on geopolymer foams based on blended aluminosilicates. For instance, Zhang et al. [10] prepared fly ash-ground granulated blast furnace slag geopolymer foam via a pre-foaming method using diluted aqueous. They concluded that the geopolymer foam based on blended precursors outperformed those produced using single precursor in terms of thermo-mechanical properties. Thus far, there are no studies on the preparation of PAS-geopolymer foams using blended precursors to investigate their performance at room temperature and high temperatures.

Consequently, the PAS-geopolymer foams produced in this study were made from a mixed precursor of fly ash and ladle furnace slag. The physical, mechanical and thermal performance of fly ash-slag blended geopolymer foams were evaluated at room temperature (29 °C) and high temperatures (200–1000 °C). The microstructural, functional group and phase analyses of fly ash-slag blended geopolymer foams were also assessed. The PAS foam-to-paste ratio was fixed at 1.0 and 2.0 to prepare geopolymer foam. Unfoamed geopolymer was also prepared to compare with foamed geopolymer. Investigation of the influence of PAS foam addition on the thermal properties of fly ash-slag blended geopolymer foam is a worthwhile effort.

## 2. Materials and Methods

### 2.1. Raw Materials

Fly ash and ladle furnace slag were mixed as a blended precursor to prepare geopolymer foam. Table 1 demonstrates the chemical composition of fly ash and slag using X-ray fluorescence. Three bulks of fly ash and slag were analysed. SiO_2_ and Al_2_O_3_ were the main compounds of fly ash, with a total amount of 84.30 wt.%, which was within the suitable composition range (83.70–87.21 wt.%) to produce geopolymers [2,11]. The low CaO content demonstrated that the fly ash was Class F fly ash based on ASTM C618. On the other hand, CaO, SiO_2_ and Al_2_O_3_ were the main compounds of slag, with a total amount of 87.19 wt.%., which was similar to other studies (73.00–83.15 wt.%) for geopolymer preparation [12,13].

**Figure 1:**
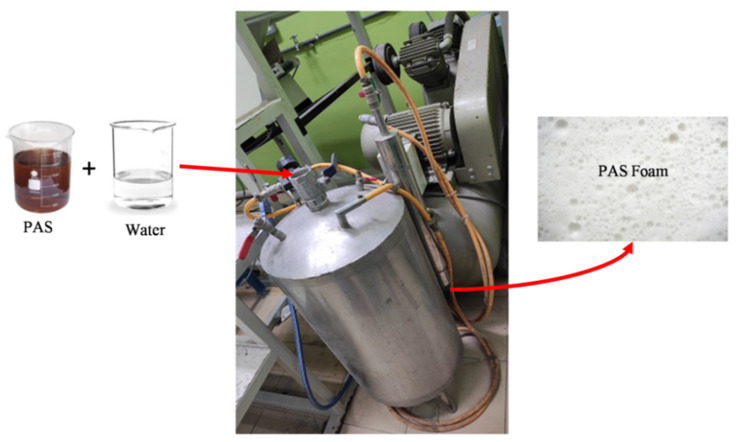
Preparation of PAS foam using a foam generator.

**Figure 2:**
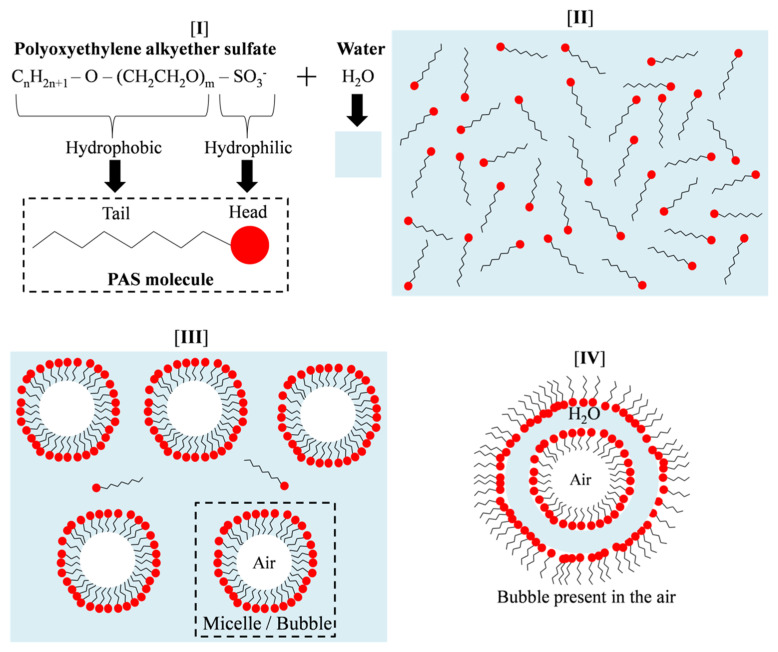
(**I**) Structural formulas of PAS and water, (**II**) PAS and water mixture, PAS foam formation in (**III**) water and (**IV**) air.

Polyoxyethylene alkyether sulphate (PAS) was utilised as a foaming agent to prepare geopolymer foam. The PAS consists mainly of 11.0 wt.% of PdO and 78.6 wt.% of SO_3_. By using the pre-foaming method, the PAS was prepared in foam before being mixed with the geopolymer mixture, as in accordance with ASTM C796. The foam was produced by PAS and water through a foam generator with a constant pressure of 0.5 MPa, as shown in Figure 1. The mechanism of PAS foam can be divided into four stages, as illustrated in a schematic diagram in Figure 2. The PAS molecule had both hydrophilic (water soluble) and hydrophobic (water insoluble) substances [14]. When PAS was mixed with water, the head of the PAS molecule favoured water while its tail resisted water. As a result, micelles formed, causing bubbles to form in the water medium. Moreover, a bubble can form in the air medium as seen in Stage IV.

Sodium hydroxide pellets (HmbG^®^, Sigma-Aldrich, Taufkirchen, Germany) and distilled water were mixed together to prepare a sodium hydroxide solution. The main constituents of the sodium silicate solution (South Pacific Chemicals Industries Sdn. Bhd., Selangor, Malaysia) were 30.1% SiO_2_, 9.4% Na_2_O, and 60.5% H_2_O. Sodium hydroxide and sodium silicate solutions were mixed to prepare an alkaline activator, and the mixed solution was placed at room temperature for 24 h before use.

### 2.2. Preparation of Unfoamed and Foamed Geopolymers

Fly ash, slag and alkaline solution were mixed together to prepare the unfoamed geopolymer, and the geopolymer was designated as G-0. The mixture was stirred with a blender (IKA-RW-20 digital, Taufkirchen,Germany) at a constant mixing speed of 1000 rpm for 15 min before being cast into a cubic (5 × 5 × 5 cm) plastic mould. The moulded mixture was placed at room temperature for 1 day, and it was further placed in an oven (60 °C) for another 1 day. The curing regime was selected in accordance to Bakharev [15], who stated that pre-curing 1 day before oven-curing for another day would lead to excellent compressive strength. The demoulded sample was stored at room temperature for 28 days before being subjected to high temperatures.

On the other hand, G-0 and PAS foam were mixed to prepare the geopolymer foam. The PAS foam-to-paste ratio was set at 1:1 and 2:1, and the PAS-to-water ratio was fixed at 1:10. The selection of these ratios was based on the optimal ratios obtained by Ibrahim et al. [5]. The foam, PAS and water were measured in volume (ml). Based on the PAS foam-to-paste ratio of 1.0 and 2.0, the resulting geopolymer foam was designated as G-1 and G-2, respectively. The geopolymer foam also used the similar curing method as the unfoamed geopolymer. The constant formulations (sodium hydroxide concentration, solid:liquid, sodium silicate:sodium hydroxide and fly ash:slag ratios) were chosen as tabulated in Table 2, owing to the optimal ratios achieved in previous work [16].

### 2.3. High Temperature Exposure

After 28 ambient-curing days, the unfoamed and foamed geopolymers were sintered to high temperatures (Table 2) in a Muffle furnace, with a heating rate of 10 °C/min and a soaking time of 2 h. The unheated geopolymer sample was also prepared as a control sample to compare with the heat-treated geopolymer sample.

### 2.4. Test and Characterisation Method

The water absorption (dry and immersion weights) and apparent porosity (dry, immersion and suspended weights) of geopolymer were measured based on ASTM C642. The bulk density (mass and volume) of geopolymer was measured in accordance with BS EN 12390-7. The volume and mass changes of geopolymer were measured before and after exposure to high temperatures.

The pore size distribution of geopolymer was determined by Synchrotron radiation X-ray tomographic microscopy, equipped with Drishti software and Octopus Reconstruction software. The specimen was prepared in a small piece (0.2 cm × 0.2 cm × 0.5 cm) for analysis.

The thermal conductivity of geopolymer was measured by a KD2 Pro Thermal Properties Analyser (Decagon Devices Inc., Pullman, WA, USA). The transient line heat source method was applied based on IEEE 442-1981 and ASTM D5334. The pre-calibration of the sensor was performed according to DB1175 before each measurement.

The compressive strength of geopolymer before and after exposure to high temperatures was tested using a mechanical tester (Instron Machine Series 5569). Three samples were tested at each exposure temperature.

The morphology of fly ash, slag and geopolymer can be visualised by a scanning electron microscope (SEM, JEOL JSM-6460 LA). Fly ash and slag were prepared in power form while geopolymer was prepared in a small piece (1 × 1 × 1 cm) for analysis. The specimens were coated with platinum using a coater (JEOL JFC 1600) before analysis.

The functional group of fly ash, slag and geopolymer was identified using Fourier transform infrared spectroscopy (FTIR, Perkin Elmer Spectrum RX1 Spectrometer), with a resolution of 4 cm^−1^ and a scan range of 650 cm^−1^ to 4000 cm^−1^. The sample was prepared in powder form for analysis.

The crystalline phase of fly ash, slag and geopolymer was carried out using an X-ray diffractometer (XRD, D2-Phaser Bruker), with 10–80° 2θ, 2°/min scan rate and 0.02° scan step. The phase patterns of specimens were analysed by X’Pert HighScore Plus software, and the specimens were prepared in powder form for analysis.

## 3. Results and Discussion

### 3.1. Effect of PAS Foam-to-Paste Ratio

**Figure 3:**
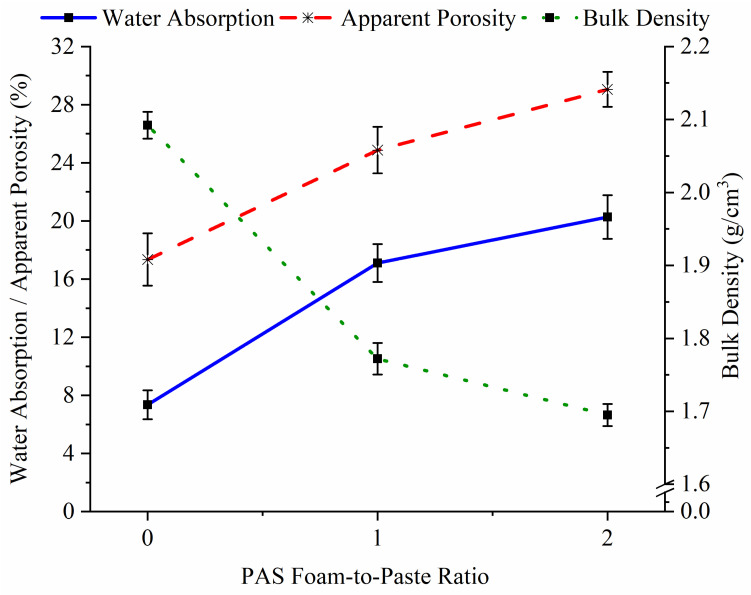
Bulk density, water absorption and apparent porosity of G-0, G-1 and G-2.

The bulk density of geopolymer can be determined by the changes in water absorption and apparent porosity, as shown in Figure 3. Geopolymer with higher porosity was expected to exhibit higher water absorption but lower bulk density. G-0 had a bulk density of 2.09 g/cm^3^, water absorption of 7.4% and apparent porosity of 17.3%. The inclusion of PAS foam increased the water absorption and porosity to 17.1–20.3% and 27.9–29.1%, respectively, while reducing the bulk density to 1.69–1.77 g/cm^3^ in geopolymer foam. When the PAS foam was added to the geopolymer, the pores in the sample would increase directly due to the increased bubbles caused by the PAS foam addition (Figure 1), subsequently reducing the bulk density. A similar explanation was observed by Ibrahim et al. [5], who reported that the bulk density of fly ash geopolymer foam decreased from 1.65 g/cm^3^ to 1.20 g/cm^3^ with increasing PAS foam content. The bulk density of the fly ash-slag blended geopolymer foam achieved in this work was greater than in their work, which was due to the high-density slag addition [16].

**Figure 4:**
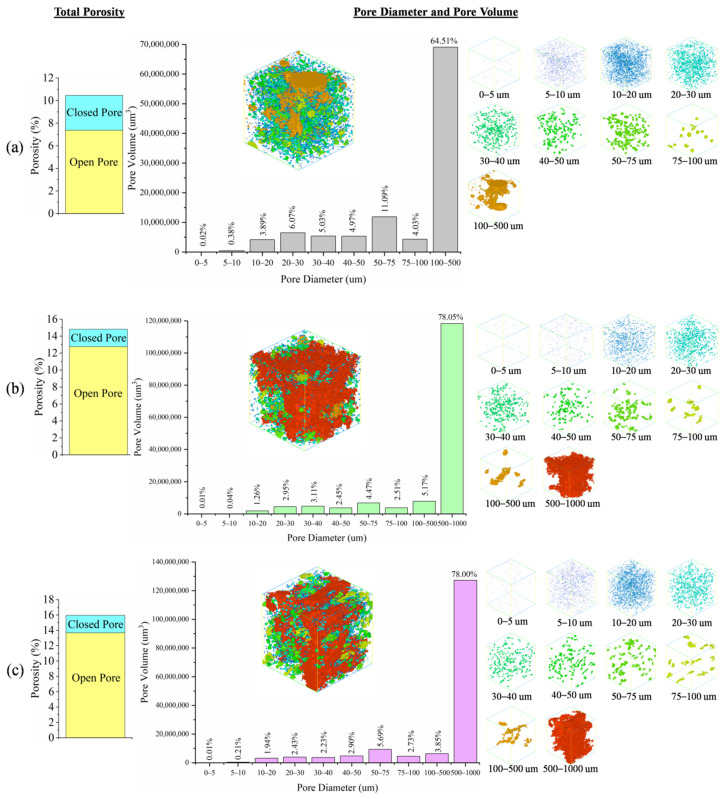
Total porosity and pore size distribution of (**a**) G-0, (**b**) G-1 and (**c**) G-2.

The pore size distribution and total porosity of G-0, G-1 and G-2 are plotted in Figure 4. G-0 (Figure 4a), G-1 (Figure 4b) and G-2 (Figure 4c) were mostly made up of open pores, while minorly made up of closed pores. However, foamed geopolymer (14.81% for G-1 and 15.94% for G-2) exhibited a greater total porosity than G-0 (10.45%), supporting the bulk density and apparent porosity results (Figure 3). This was because of the large pore diameter of 500–1000 µm (~78%) present in the foamed geopolymers but absent in G-0.

**Figure 5:**
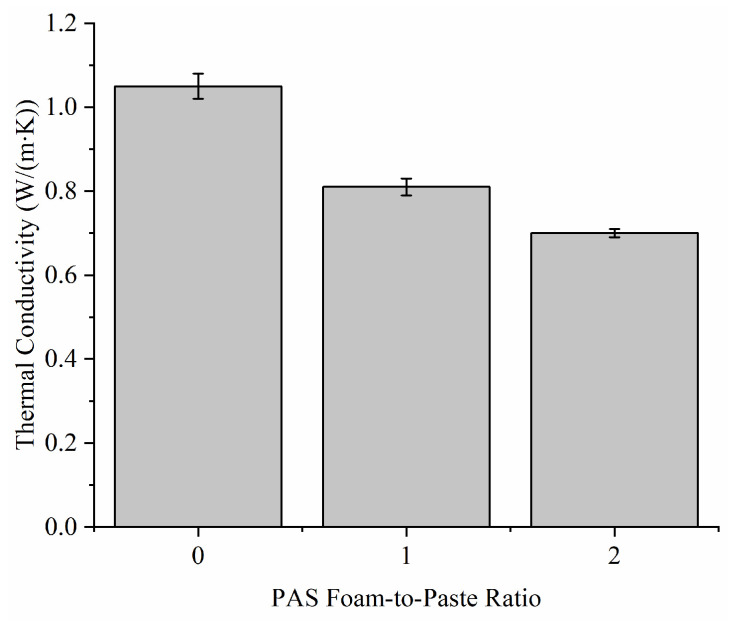
Thermal conductivity of G-0, G-1 and G-2.

The addition of PAS foam decreased the thermal conductivity of G-0 from 1.05 W/(m∙K) to 0.70–0.81 W/(m∙K), as demonstrated in Figure 5. The results were complied with bulk density and apparent porosity values, as shown in Figure 3. When a geopolymer has a higher porosity and a lower density, its thermal conductivity is always lower [17]. The trend was further supported by pore size distribution results (Figure 4) as the foamed geopolymers had larger pore sizes up to 1000 µm but G-0 had lower pore sizes up to 500 µm.

**Figure 6:**
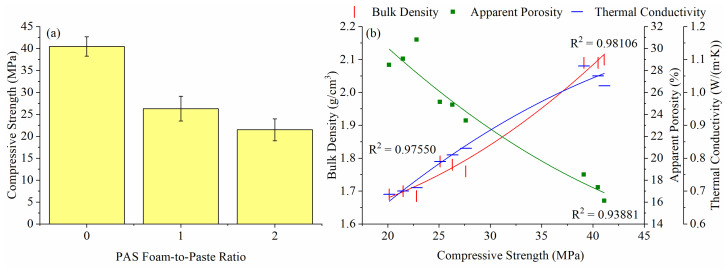
(**a**) Compressive strength and (**b**) correlation curve between compressive strength, bulk density, apparent porosity and thermal conductivity of G-0, G-1 and G-2.

The relationship between compressive strength, bulk density, apparent porosity and thermal conductivity of G-0, G-1 and G-2 is displayed in Figure 6. The addition of PAS foam decreased the compressive strength of G-0 from 40.5 MPa to 26.3 MPa (G-1) and 21.5 MPa (G-2), as shown in Figure 6a. As stated earlier, pores were induced in the geopolymer after adding PAS foam, thereby weakening the sample structure and resulting in poor strength. The explanation was further supported by Figure 6b, as the compressive strength had a strong correlation with apparent porosity, bulk density and thermal conductivity, with correlation coefficients (R^2^) of 0.93881, 0.98106 and 0.97550, respectively.

According to the functional classification of International Union of Laboratories in Construction Materials, Systems and Structures (RILEM), a sample with a density of 1.44–1.84 g/cm^3^, compressive strength of >17.0 MPa and thermal conductivity of 0.4–0.7 W/(m∙K) can be used as a structural lightweight concrete [17]. Based on the compressive strength, density and thermal conductivity values achieved in this work, only G-2 (compressive strength of 21.5 MPa, density of 1.69 g/cm^3^ and thermal conductivity of 0.7 W/(m∙K)) met the RILEM requirements for the application.

The compressive strength of the fly ash-slag blended geopolymer foam achieved in this work (21.5–26.3 MPa) was higher than the work done by Ibrahim et al. [5], who adopted the same mixing ratios of PAS foam-to-paste and PAS-to-water as in this work to produce fly ash geopolymer foam (10.2–15.8 MPa). The inclusion of slag promoted a combined matrix of calcium (CASH) and sodium (NASH) aluminosilicate hydrate in the blended geopolymer foam, while fly ash geopolymer foam only had a NASH matrix [10], thereby obtaining higher compressive strength in the blended geopolymer foam compared to the pure geopolymer foam.

### 3.2. High Temperature Performance

**Figure 7:**
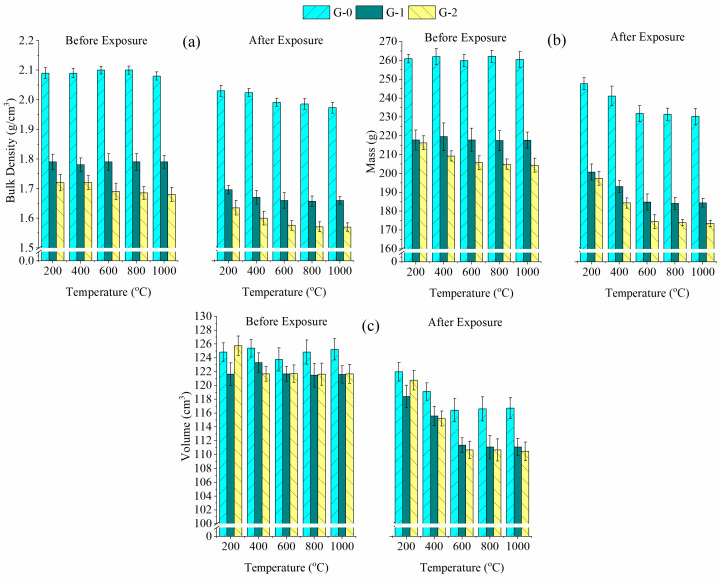
(**a**) Bulk density, (**b**) mass and (**c**) volume of G-0, G-1 and G-2 before and after exposure to high temperatures.

Figure 7 demonstrates the mass, volume and bulk density of G-0, G-1 and G-2 before and after exposure to high temperatures. Increasing temperature caused the bulk density of all geopolymers to gradually reduce up to 600 °C, and the bulk density was relatively stable between 600 °C and 1000 °C (Figure 7a). The changes in bulk density were varied by the mass and volume. The mass (Figure 7b) and volume (Figure 7c) of all geopolymers were reduced with increasing temperature up to 600 °C, and the mass and volume remained almost constant above 600 °C. The reduction in mass and volume at temperatures below 600 °C was affected by the loss of physically and chemically bonded water in the sample structure [11]. A geopolymer sample with a higher mass or lower volume was expected to have a higher bulk density.

At high temperatures, G-0 (1.97–2.03 g/cm^3^) had a higher bulk density than the foamed geopolymers (1.57–1.70 g/cm^3^). This was due to G-0 obtaining a higher remaining mass (230.25–247.60 g) and volume (116.41–121.96 cm^3^) after exposure to high temperatures compared to the foamed geopolymer (mass of 173.43–200.78 g and volume of 110.46–120.75 cm^3^). The lower residual mass and volume of foamed geopolymer were due to the increased dehydration caused by the high-moisture PAS foam addition. In terms of bulk density loss, the density loss of the fly ash-slag blended geopolymer foam in this work (4.94–7.43%) was comparatively lower than that of the fly ash geopolymer foam (17.65–28.66%) produced by Mastura et al. [2], verifying that the blended geopolymer foam showed better structural stability than the pure geopolymer foam at high temperatures.

**Figure 8:**
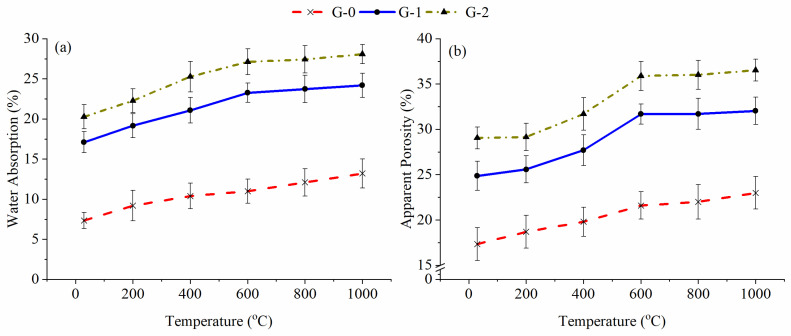
(**a**) Water absorption and (**b**) apparent porosity of G-0, G-1 and G-2 before and after being subjected to high temperatures.

G-1 and G-2 also had greater water absorption and apparent porosity than G-0 at high temperatures, as plotted in Figure 8. The porosity and water absorption of G-0, G-1 and G-2 showed parallel and similar trends, increasing from 29 °C to 1000 °C. The increased porosity from 29 °C to 600 °C was associated with the abovementioned dehydration of the sample, leading to the formation of pores in G-0, G-1 and G-2. The slight increase in porosity between 600 °C and 1000 °C might be related to the recrystallisation effect, as described in the phase analysis below. The induced new crystals caused the geopolymer matrix to slightly open and form pores in the unfoamed and foamed geopolymers.

**Figure 9:**
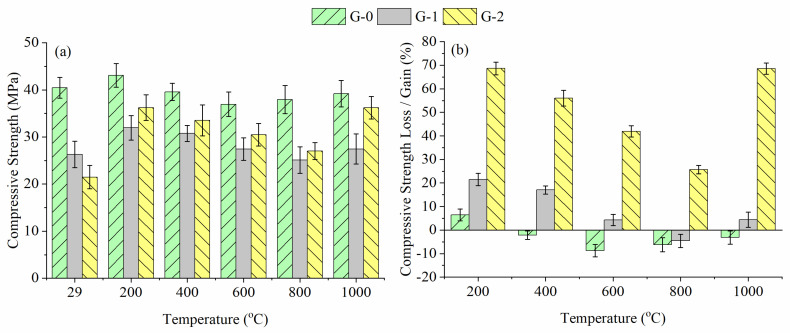
(**a**) Compressive strength and (**b**) strength changes of G-0, G-1 and G-2 with respect to unexposed samples.

Figure 9 depicts the compressive strength changes of G-0, G-1 and G-2 before and after being subjected to high temperatures. The compressive strength of all geopolymers was identical at below 600 °C, increasing from 29 °C to 200 °C and decreasing from 200 °C to 600 °C. The temperature of 200 °C caused better dissolution of slag, fly ash and alkali activator, thereby increasing the compressive strength [18]. The presence of pores (Figure 8) and bulk density loss (Figure 7) caused by the dehydration process resulted in a decrease in compressive strength at 200–600 °C. Beyond 600 °C, the trend in compressive strength of G-0 was different from that of G-1 and G-2. The strength of G-0 increased slightly at 800 °C due to the dense microstructure obtained, while the strength of G-1 and G-2 was continuously reduced at 800 °C due to the loose microstructure obtained, as shown in the following microstructural analysis. At 1000 °C, the strength of all geopolymers increased slightly was affected by phase change, as shown in the phase analysis below, which was attested by Murri et al. [13] for unfoamed geopolymer.

At high temperatures, G-0 (36.9–43.1 MPa) had a greater compressive strength than the foamed geopolymers (25.1–36.2 MPa). The explanation was consistent with the higher residual bulk density, mass and volume (Figure 7) and lower porosity (Figure 8) obtained in G-0 compared to the foamed geopolymers. The addition of PAS foam degraded the compressive strength of the control sample, resulting in the unfoamed geopolymer possessing better heat resistance than the foamed geopolymer.

However, referring to Figure 9b, G-0 lost compressive strength (2.2–8.7%) between 400 °C and 1000 °C, while foamed geopolymer gained compressive strength (4.2–68.6%) at the same temperature exposure. An exception occurred in G-1 at 800 °C due to strength loss (4.6%). As stated earlier, foamed geopolymer had a higher porosity than the unfoamed geopolymer. High porosity allowed moisture to be removed during heating without deteriorating the structure of the foamed geopolymer, while pressure was generated in the unfoamed geopolymer during the moisture removal process and resulted in a loss of strength. Therefore, the compressive strength of the foamed geopolymer at high temperatures was greatly improved due to the increased pores, especially in the case of G-2. The increase in porosity improved the thermal resistance of geopolymer foam [19]. In addition, the lower thermal conductivity value (Figure 5) of foamed geopolymer, particularly G-2, facilitated the heat transfer and reduced the damaging effect of heat at elevated temperatures.

Mastura et al. [2] heated fly ash geopolymer foam at temperatures ranging from 200 °C to 800 °C, obtaining a compressive strength in the range of 18.6 MPa to 33.3 MPa and a strength loss in the range of 29.3% to 60.4%. The fly ash-slag blended geopolymer foam produced in this work achieved better compressive strength (25.1–36.2 MPa) than their work even at high temperatures up to 1000 °C. The strength in this work even increased (4.2–68.6%) at high temperatures instead of losing strength, which was also different from their work. Thus, the geopolymer foam made with blended precursors was more stable than the geopolymer foam made with a single precursor at high temperatures.

The influence of various foaming techniques and foaming agents on the thermo-mechanical performance of geopolymer foam has been widely investigated. Geopolymer foam prepared by varying foaming methods and foaming agents resulted in different thermo-mechanical properties. For instance, Bai et al. [8] added hydrogen peroxide (H_2_O_2_) into geopolymer by adopting the direct foaming method, obtaining a compressive strength in the range of 4.5 MPa to 20.4 MPa at high temperatures. Le-ping et al. [9] used the direct foaming method to incorporate alumina powder (AP) into geopolymer, obtaining compressive strengths ranging from 6.3 MPa to 13.8 MPa at high temperatures. This work and Mastura et al. [2] work used the pre-foaming method to add PAS foam into geopolymer, with compressive strength of 18.6–36.2 MPa at high temperatures, which showed better strength and thermal performance than the geopolymer foam incorporated with H_2_O_2_ or AP. This was because PAS was an amphiprotic substance that was strongly hydrophilic and easily dissolved in water during the pre-foaming process, yielding and resulting in the formation of homogeneous air bubbles and pores [20]. However, when H_2_O_2_ or AP interacted with the geopolymer matrix, pores of varying sizes and shapes were formed during the hydrogen gas generation [3]. Thus, PAS-geopolymer foam prepared by the pre-foaming method achieved a more stable pore size and shape when compared to the H_2_O_2_- or AP-geopolymer foam prepared by the direct foaming method, resulting in PAS-geopolymer foam outperforming H_2_O_2_- or AP-geopolymer foam at high temperatures.

### 3.3. Microstructural Analysis

**Figure 10:**
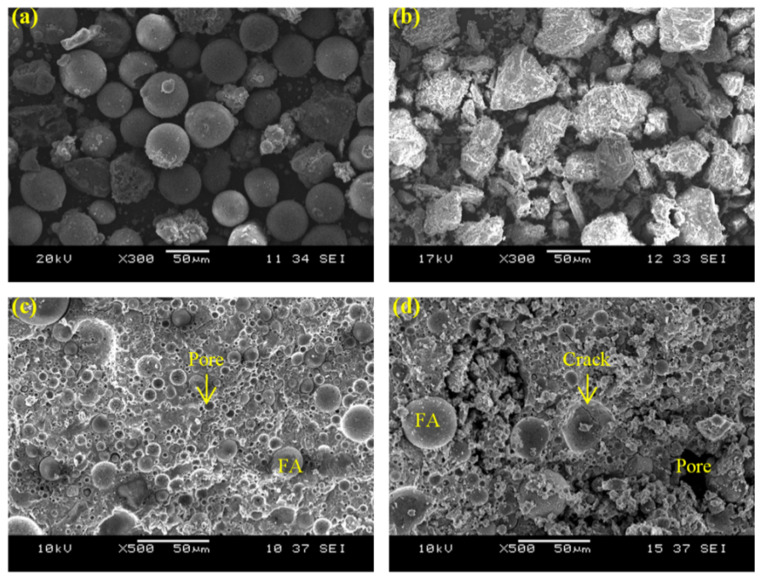
SEM images of (**a**) fly ash, (**b**) slag, unheated (**c**) G-0 and (**d**) G-2 (FA denoted fly ash).

The SEM images of fly ash and slag were taken at a magnification of × 300, as shown in Figure 10. The fly ash particles (Figure 10a) were spherical, while the slag particles (Figure 10b) were presented in block shape. On the other hand, the SEM images of unheated G-0 (Figure 10c) and G-2 (Figure 10d) were taken at a magnification of × 500. G-0 and G-2 were chosen due to the greatest difference in compressive strength between G-0 and G-2 (Figure 6). Generally, the geopolymer matrix in G-0 and G-2 showed some pores, cracks, remaining fly ash and slag particles. A more compact microstructure was observed in G-0 as compared to G-2. The larger pores and cracks of G-2 were caused by the pores induced by PAS foam addition, as supported by bulk density and porosity results (Figure 3 and Figure 4), thereby achieving a low compressive strength.

**Figure 11:**
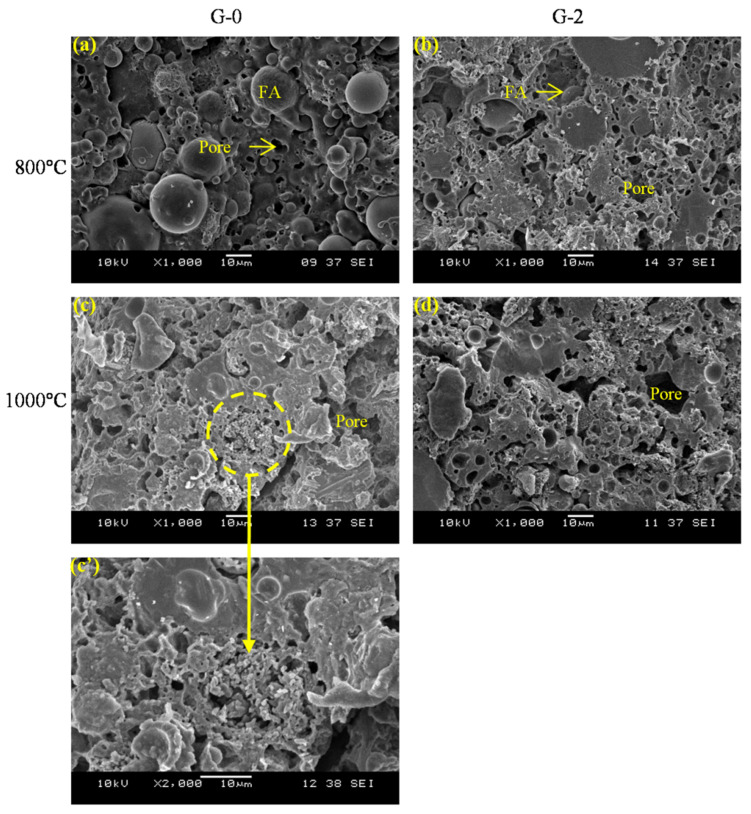
SEM images of G-0 and G-2 after exposed to (**a**,**b**) 800 °C and (**c**,**c’**,**d**) 1000 °C (FA denoted fly ash).

Figure 11 depicts the SEM images of G-0 and G-2 after being heated to 800 °C and 1000 °C. The temperatures of 800 °C and 1000 °C were selected based on the different compressive strength trends of G-0 and G-2 at 800 °C (Figure 9). At 800 °C, the geopolymer matrix in G-0 was observed to be connected and smooth with a small number of pores and residual particles (Figure 11a), leading to a slight improvement in the compressive strength. However, the compressive strength of G-2 was further degraded at 800 °C due to the loose matrix obtained with a large number of pores (Figure 11b). At 1000 °C, the disappearance of residual particles and the appearance of the solidified melt in the geopolymer matrix [21] most likely contributed to the increase in compressive strength of G-0 (Figure 11c) and G-2 (Figure 11d). The presence of rod and/or plate anorthite crystal (Figure 11c’) was also expected to enhance the compressive strength of G-0 at 1000 °C. Anorthite was typically discovered in a high-calcium geopolymer system at 1000 °C [22].

### 3.4. Phase Analysis

**Figure 12:**
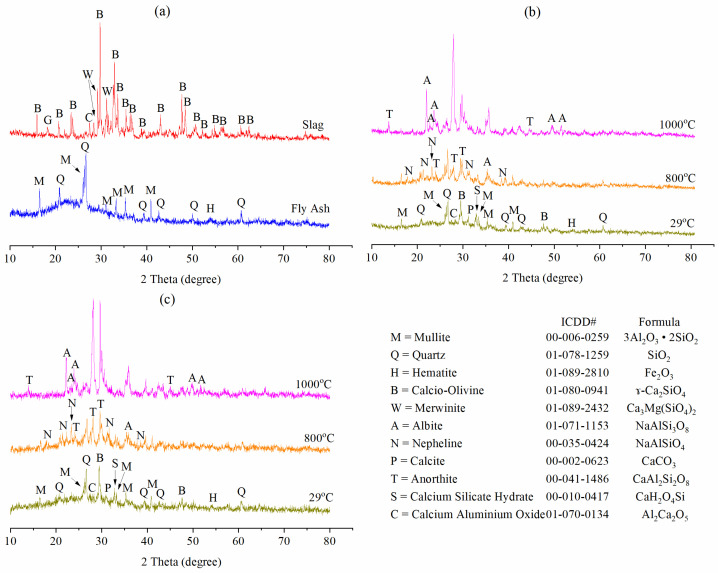
Phase patterns of (**a**) fly ash and slag, unexposed and temperature-exposed (**b**) G-0 and (**c**) G-2.

Figure 12 illustrates the phase patterns of fly ash, slag, unexposed, and temperature-exposed G-0 and G-2. Fly ash had an amorphous broad hump in the range of 15° to 35° 2θ, with some peaks of mullite, quartz and hematite. Slag was mainly crystalline with peaks of calcio-olivine, merwinite, magnetite and calcium aluminium oxide. Unheated G-0 and G-2 (Figure 12b,c) had an amorphous broad hump between 16–38° 2θ, also had some peaks of mullite, quartz, hematite, calcio-olivine and calcium aluminium oxide, which came from raw fly ash and slag. In addition, some new peaks of calcite and calcium silicate hydrate (CSH) appeared in the unheated samples, which were caused by alkali activation of fly ash and slag.

The rise in temperature caused the (i) crystalline peaks to change, increase, reduce, disappear and/or decompose and (ii) amorphous phase (geopolymer matrix) to reduce and/or decompose (Figure 12b,c). Calcite and CSH vanished and decomposed at 800 °C, as supported by Shaikh [23] for unfoamed geopolymer. The combined decomposition of the geopolymer matrix, calcite and CSH caused nepheline, albite and anorthite to form at 800 °C and 1000 °C. The presence of anorthite was also confirmed by the SEM image in Figure 11c’. The addition of PAS foam did not cause any changes to the phase patterns of geopolymer at room temperature and high temperatures.

### 3.5. Functional Group Identification

**Figure 13:**
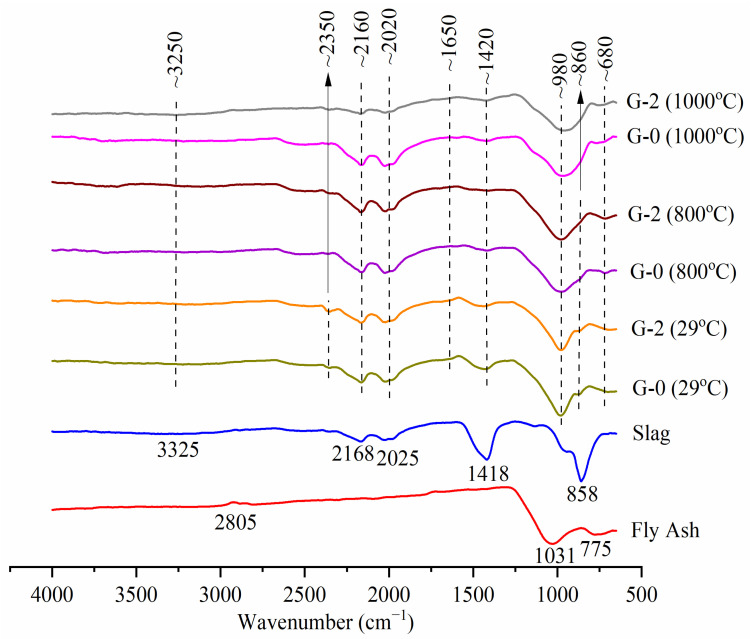
IR spectra of fly ash, slag, unexposed and temperature-exposed G-0 and G-2.

Figure 13 plots the IR spectra of fly ash, slag, unexposed and temperature-exposed G-0 and G-2. The main band of raw fly ash was located at 1031 cm^−1^, corresponding to the asymmetric stretching vibration of Si-O-X (X = Si or Al) [24]. The band at 775 cm^−1^ was expressed as the symmetric stretching vibration of Si-O-Si [25]. Moreover, the main band of raw slag was located at 858 cm^−1^, indicating the stretching vibration of Ca-O and Si-O [26]. The bands at 2168 cm^−1^, 2025 cm^−1^ and 1418 cm^−1^ were verified as the stretching vibrations of O-C-O [27]. The bands at 2805 cm^−1^ and 3325 cm^−1^ were denoted as the stretching vibrations of OH and H-O-H [28].

The presence of O-C-O (~2160, ~2020 and ~1420 cm^−1^) and OH and H-O-H (~3250 cm^−1^) stretching vibrations in the unheated G-0 and G-2 originated from raw fly ash and slag. New bands of calcite [29] and H-O-H bending vibration [30] appeared at ~2350 cm^−1^ and ~1650 cm^−1^, respectively, in unheated G-0 and G-2. Calcite disappeared and decomposed at 800 °C, as proven by the phase analysis in Figure 12. The wavenumber of fly ash at 1031 cm^−1^ and 775 cm^−1^ shifted to ~980 cm^−1^ and ~680 cm^−1^, respectively, in unheated G-0 and G-2, inferring the NASH matrix was formed [28,31]. The wavenumber of slag at 858 cm^−1^ shifted to a higher value in the unheated G-0 and G-2 (~860 cm^−1^), implying the CASH matrix was formed [32].

The rise in temperature changed the intensity of the absorption bands of G-0 and G-2. The main band at ~980 cm^−1^ widened at high temperatures, especially at 1000 °C, indicating that the high temperatures led to the formation of structural disorder, as proven by the literature for unfoamed geopolymer [33,34]. The intensity of ~1650 cm^−1^ reduced at high temperatures, inferring that the geopolymer had dehydrated [19]. The intensity at ~860 cm^−1^ vanished at 1000 °C, implying that the cross-linking in the CASH matrix was lessened [35]. The addition of PAS foam did not create any new bands in the geopolymer at room temperature and high temperatures.

## 4. Conclusions

This paper compares the thermo-mechanical performance of fly ash-ladle furnace slag blended geopolymer with and without PAS foam addition at 29–1000 °C. The PAS foam-to-paste ratio of 1.0 and 2.0 was used to prepare geopolymer foam. The incorporation of PAS foam degraded the compressive strength of unheated G-0 (40.5 MPa) by 35.0–46.9%, and also decreased the heat-treated G-0 (36.9–43.1 MPa) by 7.6–33.9%. Nonetheless, when compared to unheated samples, the compressive strength of the heat-treated G-0 decreased by 2.2–8.7% at 400–1000 °C, whereas the compressive strength of the heat-treated G-1 and G-2 increased by 4.2–68.5% at the same temperature exposure. The higher porosity, lower thermal conductivity and lower connectivity of the geopolymer matrix aided dehydration and reduced deterioration of the geopolymer structure, resulting in a significant improvement in the strength loss of foamed geopolymer.

## Figures and Tables

**Table 1:** Chemical composition of fly ash and slag.

Compound	Fly Ash (wt.%)	Slag (wt.%)
SiO_2_	56.30 ± 0.15	21.30 ± 0.10
Al_2_O_3_	28.00 ± 0.30	2.30 ± 0.20
CaO	3.89 ± 0.16	63.59 ± 0.13
Fe_2_O_3_	6.86 ± 0.05	8.08 ± 0.12
K_2_O	1.49 ± 0.04	-
MgO	-	2.60 ± 0.20
TiO_2_	2.17 ± 0.04	0.50 ± 0.16
PdO	-	-
SO_3_	-	-
Others	1.29 ± 0.16	1.63 ± 0.09

**Table 2:** Details of sample preparation.

Category	Unfoamed Geopolymer	Foamed Geopolymer	
Abbreviation	G-0	G-1	G-2
PAS Foam:Paste Ratio	0:0	1:1	2:1
Fly ash:Slag Ratio	80:20		
Sodium Hydroxide Concentration (M)	8		
Solid:Liquid Ratio	3:1		
Sodium Silicate:Sodium Hydroxide Ratio	1.5:1		
PAS:Water Ratio	1:10		
Exposure Temperature (°C)	29, 200, 400, 600, 800 and 1000		

## Data Availability

All the data is available within the manuscript.
